# Supplementary material for: Are carbon emissions trading and green financial instruments synergistic? -Comprehensive quantitative research based on content analysis
Source: PLoS One. 2024 Mar 7;19(3):e0298601. doi: 10.1371/journal.pone.0298601 (PMC10919601; doi:10.1371/journal.pone.0298601)
Supplement: S1 Data — (ZIP) [file pone.0298601.s001.zip › Data/地级市Z_F工作报告.zip!/╜¡╦╒/╬▐╬2╩╨/└┤╫╘╣1⁄2╓┌║┼í╛┬φ┐╦╩2╛▌═°í┐_2019.docx]

各位代表：

　　现在，我代表无锡市人民政府，向大会报告工作，请予审议，并请市政协委员和列席人员提出意见。

　　一、2018年工作回顾

　　过去一年，市政府以习近平新时代中国特色社会主义思想为指导，认真贯彻落实党的十九大精神和习近平总书记对江苏工作的重要指示要求，在中共无锡市委的坚强领导下，在市人大、市政协的监督和支持下，紧紧依靠全市广大人民，按照当好全省高质量发展领跑者的目标定位，坚持稳中求进工作总基调，深入践行新发展理念，坚定实施六大发展战略，较好完成了市十六届人大二次会议确定的目标任务，为决胜“十三五”后半程打下了坚实基础。

　　（一）经济运行稳中有进。坚决落实中央和省、市委决策部署，扎实做好稳就业、稳金融、稳外贸、稳外资、稳投资、稳预期工作，经济保持平稳健康发展。15个主要经济指标中，10个指标增幅高于全省平均。全市地区生产总值突破11000亿元，达到11438.62亿元，增长7.4%，增幅跃居全省第二；　规模以上工业增加值达到3618.71亿元，增长9%，增幅20年来首次跃居全省第一；一般公共预算收入跨上千亿元台阶，达到1012.28亿元，增长8.8%，其中税收收入占比达到85%。社会融资规模累计新增1669.73亿元。有效投资持续扩大。固定资产投资增长5.8%，其中工业投入增长10.5%、民间投资增长10.7%。100个市级重大项目完成投资978.5亿元，超过年度计划12.8个百分点。华虹集成电路、中环大硅片、恩捷新材料等项目开工建设，领途汽车、村田锂电池新工厂等项目竣工投产，远景日产智能电池、闻泰科技5G智能终端等项目签约落地。消费需求稳定增长。社会消费品零售总额完成3672.7亿元，增长9%，消费满意度测评全国第一。外贸进出口创历史新高。主动应对中美经贸摩擦，帮助企业巩固和开拓市场，实现进出口总额934.44亿美元，增长15%，其中出口总额567.81亿美元，增长14.7%。成功获批国家跨境电商综合试验区，成为全省唯一的国家文化出口基地，圆满完成首届中国国际进口博览会参展任务。企业支撑作用增强。全市净增规模以上企业1721家，其中工业企业570家，均位居全省第一。69家企业入选2018中国企业500强等4个“500强”榜单，比上年增加9家，占全省入围数的30%，继续位居全省第一。新增境内外上市公司11家，总量保持全省第一。中信泰富特钢成为全市第二家营业收入超千亿元的企业。经济增长体现更多民生含量。新增城镇就业16.14万人，城镇登记失业率控制在1.78%。预计居民人均可支配收入增长8.4%左右，高于同期经济增长速度。居民消费价格上涨控制在3%之内。

　　（二）产业强市成效明显。新兴产业发展势头良好。规模以上战略性新兴产业（制造业）增加值增长13.9%。积极筹建国家物联网创新中心，成功举办2018世界物联网博览会，海尔物联生态网基地等重大项目签约落户，车联网等应用示范项目加快推进。加大集成电路产业支持力度，深化与国家集成电路产业投资基金的战略合作，无锡国家集成电路设计产业园揭牌，国家芯火双创基地平台成功获批。传统产业智能化步伐加快。举办首届雪浪大会，启动“千企上云”行动，100个智能制造重点项目建设进展顺利，4个项目入选工信部智能制造示范应用项目，1个项目入选工信部工业互联网试点示范项目，新增省级示范智能车间36家。军民融合公共服务平台启动建设，富华高技术研究院等重点项目成功落地。现代服务业发展提质增效。新认定市级总部企业5家，SK海力士中国销售总部落户无锡。推进国家全域旅游示范区创建，鸿山旅游度假区跻身省级旅游度假区，海澜飞马水城正式开园，全市旅游总收入增长11.5%，游客满意度连续四年保持全省第一。成功举办中国（无锡）国际新能源大会等会展活动142个。科技创新能力不断增强。落实全市科技创新与人才大会精神，组织实施“创新10条”，预计全社会研发投入占地区生产总值比重达到2.85%，企业研发经费占销售收入比重达到1.73%，科技进步贡献率达到63.9%，万人有效发明专利拥有量达到38件，继续保持全省领先。实施“创新型企业倍增计划”，预计新增高新技术企业395家，高新技术产业产值占规模以上工业产值比重达到43.3%，10家企业入选省科技小巨人企业名单。启动与驻锡科研院所“一所一策”合作。中比摩蝎（欧洲）科技创新产业投资基金、太湖新兴产业成长基金签约落地。实施“太湖人才计划”升级版2.0，新增各类人才7.49万人、院士工作站12家。节约集约发展成效明显。预计单位建设用地GDP产出提高到7.27亿元/平方公里、税收收入提高到7.24万元/亩，区域建设用地节约集约利用综合指数排名全省第一。

　　（三）改革开放协同发力。供给侧结构性改革深入推进。多措并举降低企业成本200亿元以上，全市银行业金融机构不良贷款余额和不良贷款率连续五年实现“双下降”，地方政府债务余额有效控制在限额范围内，市区非住宅商品房库存同比减少17.65万平方米。重点领域和关键环节改革步伐加快。“放管服”改革持续深化，赋予江阴市282项行政权力、6家省级以上开发区187项市级经济管理权限，建成覆盖全市的“互联网+政务服务”体系，市区不动产登记实现“一窗综合受理、一网全市通办、一个小时办结”，全年新登记企业5.16万户。行政体制改革成效明显，江阴市县级集成改革试点经验在全省复制推广，第二批省级经济发达镇改革正式启动，太湖新城管理体制优化调整基本到位，滨湖区全国基层政务公开标准化规范化试点工作圆满完成。经济体制改革不断深化，国税地税征管体制改革推进顺利，国发资本运营有限公司正式组建，申报设立锡商民营银行取得积极进展。户籍制度改革红利进一步释放，5.6万人落户无锡。对外开放水平进一步提高。赴以色列、日韩、欧洲等地开展经贸招商活动，完成到位注册外资37.1亿美元。江阴综合保税区（一期）顺利通过国家验收，无锡高新区综合保税区增值税一般纳税人资格试点成效明显。积极参与“一带一路”建设，预计完成1000万美元以上重大境外投资项目23个，柬埔寨西港特区入驻企业达到153家，埃塞俄比亚纺织基地建设进展顺利。苏南硕放机场完成旅客吞吐量721万人次、增长7.8%，货邮吞吐量12.4万吨、增长15.1%，洲际货运航班频次位列全省第一。药品进口口岸通过国家级评估验收。成功举办第九届无锡市国际友城交流会、第五届国际赏樱周等外事活动，荣获“国际友好城市交流合作奖”。

　　（四）城乡区域协调发展。锡澄锡宜一体化取得新进展。南沿江铁路开工建设，苏锡常南部高速进入太湖隧道主体结构施工阶段，锡澄城际轨道S1线完成工可报告编制，宜兴丁蜀通用机场获批立项，340省道无锡段改扩建等项目开工建设。城市建设管理水平得到新提升。主动融入长江经济带高质量发展大局，积极呼应长三角一体化发展国家战略，新一轮城市总体规划编制形成阶段性成果，宜兴市城市总体规划获省政府批复。梁溪区八大片区棚改全面启动，太湖新城、锡东新城、惠山新城综合功能进一步完善。无锡火车站一体化改造工程竣工投用。地铁1号线南延线全线“电通”，3号线一期全线“洞通”，4号线一期进入盾构施工。蠡湖大道、江海西路快速化改造工程及具区路、吴桥东路等重点道桥建成通车。新沟河拓浚延伸工程基本完成。全市林木覆盖率达到27.3%以上，自然湿地保护率达到54%以上。制定出台新型智慧城市建设三年行动计划，无锡入选“全球智慧城市20强”榜单。落实全市城市管理工作会议精神，启动优美环境合格区建设，开展七大专项提升行动和“小飞龙”专项整治，制定市区停车泊位三年建设计划，完成30条主要道路包装出新和30个背街小巷综合治理，拆除违法建设69.6万平方米。大力实施乡村振兴战略。落实全市乡村振兴大会精神，十大工程建设取得良好开局，成功举办首届“中国农民丰收节”江苏主场活动和现代农业博览会等重大活动。全市种植业绿色优质农产品面积比重达到60%，首个市级农产品地理标志“无锡毫茶”通过国家认定。实施“百企建百园”工程，新增省级农业产业化龙头企业5家、国家级示范合作社7家、省级示范合作社11家、省级示范家庭农场16家。聚焦农村发展短板，启动农村住房更新改造首批试点工作。阳山镇成功入围中央农业产业强镇示范建设名单，璜土镇获评全国“一村一品”示范镇，斗山村获评中国美丽休闲乡村。

　　（五）环境质量持续向好。“减煤”“减化”任务超额完成。整治燃煤锅炉102台、燃煤工业窑炉34座，减少煤炭消费总量138.5万吨，预计全市单位GDP能耗下降4%以上。扎实推进化工企业“四个一批”专项行动，关停化工企业250家，升级改造373家。组织实施261项太湖治理和114项水污染防治重点工程项目，完成沿太湖3公里缓冲带水产养殖整治和滆湖围网拆除工作，全面加强河道环境综合整治，基本完成城市黑臭水体治理任务，45个国省考断面优III比例提升到64.4%，重点水功能区水质达标率稳步提升，太湖无锡水域主要水质指标好于全太湖，连续第十一年实现安全度夏和“两个确保”。组织实施1208项大气污染防治重点工程项目，开展挥发性有机物污染治理专项行动，全市PM2.5平均浓度下降到43微克/立方米，空气优良天数比率提高到70.7%，大气污染防治年度考核综合评分全省第一。推进4个重金属重点防控区专项整治，完成土壤修复项目7个。固危废处置能力稳步提升，惠联餐厨废弃物处理厂、蓝藻藻泥和市政污泥处置项目启动建设，益多环保大修项目如期复产，江阴秦望山危废集中焚烧处置设施、宜兴凌霞危废焚烧扩建项目顺利建成。开展“绿刃”环保专项行动，关停取缔“散乱污”企业（作坊）8106家，整治完成5475家。环保督察整改任务全面落实。中央环保督察整改工作基本完成，“清废2018”通报问题实现整改销号，县级饮用水水源地环境问题提前完成整改，中央环保督察“回头看”、太湖治理督导、长江经济带生态环保审计、省环保督察反馈问题整改工作扎实推进。

　　（六）人民生活不断改善。市本级公共财政支出的80%投向民生，10件为民办实事项目全部完成，基本公共服务体系建设效果满意度全省第一。社会保障水平稳步提高。“阳光扶贫”覆盖全市2.36万户、3.45万人，市区最低生活保障标准提高到每人每月900元，26个经济薄弱村实现脱困转化。职工基本养老保险扩面19.02万人，医疗保险扩面17.81万人，失业保险扩面12.21万人，工伤和生育保险均扩面15.88万人。市区企业退休人员人均养老金提高到每月2747元，城乡居民基础养老金增加到每月450元，被征地农民政府保养人员保养金每月增加90元。完成棚户区改造51.3万平方米、旧住宅区整治220万平方米。社会事业加快发展。落实全市教育大会精神，完成中小学、幼儿园基建项目35个，新建4所省现代化示范性（优质特色）职业学校，完成1158家学科类校外培训机构集中整治。与东南大学签署新一轮市校合作共建框架协议，南京信息工程大学滨江学院无锡校区正式招生，南京理工大学江阴校区、江南大学宜兴研究生院开工建设，江南大学江阴校区正式签约，无锡太湖学院获批硕士学位立项建设单位。落实全市卫生与健康大会精神，深化医药卫生体制改革，实施市属医疗卫生机构布局调整优化方案，锡山人民医院新院区建成投用，17家基层医疗卫生机构完成提档升级，新建一批医疗技术协作中心，顺利通过国家卫生城市复审评估。深入推进全国养老服务业综合改革试点、全国居家和社区养老服务改革试点，启动儿童福利院重建和救助站、军供站搬迁工作，“慈福”民生保险项目获评“中华慈善奖”。成功承办首届江南文脉论坛，惠山古镇二期开工建设，启动龙光塔等文物修缮工程46项，建成基层综合性文化服务中心471个，歌剧《二泉》入选“全国优秀歌剧巡演”，舞剧《英雄·玛纳斯》获“丝路文化贡献奖”。成功举办2018世界击剑锦标赛，成功申办2021世界跆拳道锦标赛，无锡国际马拉松晋级国际田联“铜标赛事”，我市代表团参加第19届省运会取得历史最好成绩。社会保持和谐稳定。加强和完善城乡社区治理，全市98%的城市社区、97%的农村社区达到省级和谐社区建设标准。诚信无锡建设深入推进，公共信用信息服务基本实现全覆盖。第四次全国经济普查全面展开。扎实推进安全生产领域改革发展，积极创建国家食品安全示范城市。“雪亮工程”一期竣工投用，扫黑除恶专项斗争取得明显成效，公众安全感连续三年保持全省第一。积极支持国防和军队改革，认真做好国防动员、双拥共建、优抚安置、人民防空等工作。信访、史志、档案、外事、对台事务、港澳、侨务、民族宗教、气象、防震减灾、对口帮扶等工作取得新成绩，妇女、儿童、老龄、残疾人、红十字等事业有了新发展。

　　过去一年，面对艰巨繁重的改革发展稳定任务，市政府深入学习贯彻习近平新时代中国特色社会主义思想和党的十九大精神，严格落实全面从严治党主体责任，坚持依法行政，大力转变工作作风，政府自身建设取得新进展新成效。积极开展解放思想大讨论活动，深入基层加强调查研究，有效解决一批企业和群众反映突出的问题。落实法治政府建设规划，提请市人大常委会制定地方性法规4件，出台市政府规章4件，新聘任市政府法律顾问10名。进一步优化和完善市政府工作制度，自觉接受市人大、市政协及各民主党派的监督，市人大代表议案、建议和政协委员提案办理满意率均创五年来新高。抓好巡视巡察反馈问题整改，认真执行中央八项规定及其实施细则精神和省、市委具体办法，市本级“三公”经费支出下降3.9%。加强对重大决策部署落实情况和公共资金、公共资产、公共资源的审计监督，市本级审计发现问题实现整改全覆盖。充分运用鼓励激励、容错纠错、能上能下“三项机制”，努力营造想干事、能干事、干成事的浓厚氛围。

　　各位代表，这些成绩的取得，是中共无锡市委坚强领导的结果，是市人大、市政协大力支持和加强监督的结果，也是全市广大干部群众继往开来、众志成城、共同奋斗的结果。在此，我代表无锡市人民政府，向全市人民，向各位市人大代表、政协委员，向市各民主党派、工商联、无党派人士，向各人民团体、各界人士，向驻锡人民解放军、武警官兵和公安干警，向所有关心和支持无锡建设发展的海内外朋友，表示衷心的感谢并致以崇高的敬意！

　　在肯定成绩的同时，我们也清醒地看到，无锡经济社会发展还存在一些困难和问题，政府工作也存在一些不足。主要是：经济下行压力有所加大，经济发展的不确定性因素增多，持续向好的基础还不稳固；科技创新力度有待加大，新动能、新技术、新业态支撑能力还不强；在生态环保、基础设施、民生改善、城市管理等领域还存在一定短板；政府服务效能需要进一步提高，少数干部服务企业和群众不够积极主动，办事拖拉、敷衍塞责、缺乏担当等问题依然存在，等等。对此，我们将进一步增强忧患意识，突出问题导向，在今后工作中采取切实有效的措施认真加以解决。

　　二、2019年主要工作

　　今年是新中国成立70周年，也是决胜高水平全面建成小康社会的关键之年，做好今年工作至关重要。当前，世界面临百年未有之大变局，但和平与发展的时代主题没有改变，经济全球化的趋势没有改变，我国发展仍处于并将长期处于重要战略机遇期的判断没有改变，变局中危和机同生并存，需要我们进一步认识规律、尊重规律、把握规律、运用规律，强化辩证思维、系统思维、聚焦思维、底线思维，以改革开放再出发的信心和勇气，恪尽职守、毫不懈怠，开拓创新、奋力攻坚，努力交出一份人民满意的高质量发展答卷。

　　根据中共无锡市委十三届七次全会的部署，今年政府工作的总体要求是：以习近平新时代中国特色社会主义思想为指导，深入贯彻党的十九大精神，统筹推进“五位一体”总体布局，协调推进“四个全面”战略布局，按照中央经济工作会议和省委十三届五次全会部署，坚持稳中求进工作总基调，坚持新发展理念，坚持推动高质量发展，坚持以供给侧结构性改革为主线，坚持深化市场化改革、扩大高水平开放，坚定实施六大战略，坚定打好三大攻坚战，坚定推进全面从严治党，努力实现创新活力和转型动力更强，经济运行和发展态势更稳，生态质量和人民生活更好，政治生态和干事环境更优，打好高水平全面建成小康社会决定性基础，夺取“强富美高”新无锡建设关键性胜利，奋力当好全省高质量发展领跑者，以优异成绩迎接新中国成立70周年。

　　综合各方面因素，今年全市经济社会发展主要目标是：地区生产总值增长7%以上；规模以上工业增加值增长8%左右；一般公共预算收入增长6%左右；固定资产投资增长6%左右；社会消费品零售总额增长9%左右；外贸进出口稳中提效；　全社会研发投入占地区生产总值比重达到2.87%；城乡居民收入增长与经济增长同步，城镇登记失业率、居民消费价格涨幅控制在省定范围内；　节能减排和大气、水环境质量确保完成省下达的目标任务。

　　各位代表，做好今年工作，我们将准确把握党中央对新形势下做好经济工作“五个必须”的规律性认识，深入贯彻落实中央经济工作会议和省委十三届五次全会、市委十三届七次全会精神。一是坚定实施创新驱动核心战略和产业强市主导战略。保持产业强市的战略定力，把经济发展的着力点放在实体经济上，突出现代产业发展的优先选项、主攻方向和重要取向，加快构建自主可控的现代产业体系和开放高效的产业科技创新体系，促进多种所有制经济共同发展，为推动经济发展质量变革、效率变革、动力变革蓄势赋能。二是坚决有力打好三大攻坚战。坚持问题导向，突出工作重点，打好关键战役，推动防范化解重大风险、精准脱困、污染防治工作取得突破性进展，确保不发生系统性区域性风险，确保高水平全面建成小康社会路上一个不掉队，确保生态环境质量持续向好。三是扎实推进城乡区域协调发展。充分发挥“一带一路”建设、长江经济带发展和长三角一体化发展等国家战略在无锡叠加的优势，加快推进锡澄锡宜一体化发展，促进市域范围内资源要素的深度整合与互动，激发各板块各片区的比较优势和发展潜力，在落实国家战略中提升无锡城市能级。四是持续增强人民群众的获得感幸福感安全感。坚持把保障和改善民生作为政府一切工作的出发点和落脚点，按照“劳有丰酬、弱有多扶、学有优教、病有良医、老有颐养、住有宜居”的目标，既尽力而为，又量力而行，精准对接人民群众对美好生活的需要，不断增加优质公共产品和公共服务的有效供给，确保全市人民安居乐业、社会大局安定有序。

　　重点做好以下八个方面的工作：（一）着力深化改革扩大开放

　　促进实体经济健康发展。严格执行国家和省减税降费政策，落实好33条降本减负新举措，清理规范涉企收费，切实提高企业的感受度。更大力度开展“稳心留根”和“亲商暖企”行动，全力打造有竞争力的市场化、法治化、国际化营商环境，与广大企业同舟共济、共克时艰。落实习近平总书记在民营企业座谈会上的重要讲话精神，在市场准入、经营运行、招投标等方面为民营企业创造公平竞争环境，着力缓解民营企业特别是高成长性企业的资金周转压力，依法保护民营企业家合法权益，推进锡商民营银行筹建工作，办好第四届全球锡商大会。优化调整国有资本布局结构，积极发展混合所有制经济，推进市属国有企业股份制改革，推动市属国有资本进一步向基础性、公益性领域和战略性新兴产业集聚。实施大企业大集团和“专精特新”小巨人企业培育工程，新增境内外上市公司8家。落实地方金融监管责任，持续做好重点地区、重点领域的金融风险管控化解工作，不良贷款率继续低于全省平均水平。

　　增创体制机制新优势。按照“宽放、善管、优服”的要求，纵深推进“放管服”改革，做好无锡经济开发区赋权（委托）工作，市（县）区和有条件的省级以上开发区成立行政审批局；深化综合行政执法体制改革，推进“双随机、一公开”监管全覆盖，实现“一支队伍管执法、一套清单管权责、一个中心管指挥、一个网格管治理、一个平台管信用、一套机制管检查”；全面推进“证照分离”改革，优化完善税收共治体系，建立开办企业“一网通”服务平台，实施全流程、全领域工程建设项目审批改革。统筹推进镇（街）管理体制改革，复制推广江阴市县级集成改革试点经验，落实第二批省级经济发达镇改革任务，加快构建简约高效的基层管理体制。深化诚信无锡建设，力争创成全国社会信用体系建设示范城市。

　　构筑全面开放新格局。实施外贸市场多元化战略，提高贸易通关便利化水平，加大对重点出口行业和外贸企业的扶持力度。加快国家跨境电商综合试验区建设和综合保税区升级工作，深化国家服务外包示范城市和文化出口基地建设，主动承接2019年中国国际进口博览会溢出效应。加大外资引进力度，推动现有外资企业增资扩股和提档升级，积极引进外资总部及功能性机构，确保到位注册外资36亿美元。加快开发区转型升级，优化开发区空间布局和资源配置，推动开发区综合发展水平排位稳步提升，支持惠山经济开发区、江阴临港经济开发区申报国家级开发区。积极融入全省“一带一路”交汇点建设大局，在高水平“引进来”的同时，支持企业“走出去”发展，打造柬埔寨西港特区升级版2.0，维护企业海外合法权益。推动锡台两地全方位交流，为台胞提供与无锡居民同等待遇。

　　（二）着力推进产业强市建设

　　强化重大项目支撑力。全年安排市级重点项目100个，总投资超4700亿元，当年完成投资880亿元。完善重大产业项目挂钩联系制度，推动LG汽车动力电池正极材料、深海空间站无锡研发基地等重大项目开工建设，对市（县）区重点项目实行分级调度、协同推进。加大项目招引市级统筹力度，建好重大项目“蓄水池”。深入开展工业企业资源利用绩效评价，进一步完善“亩产论英雄”综合评价和差别化资源要素配置机制。积极推进城乡建设用地增减挂钩，加大节约集约用地力度，实现重大产业项目用地需求应保尽保。

　　提升现代产业竞争力。培育发展16个先进制造业重点产业集群，加大对5G、人工智能、石墨烯、增材制造等未来产业的布局力度，推动产业加快迈向中高端。完成现代产业发展三个“三年行动计划”年度目标任务，积极推进智能交通、智慧健康等园区建设，提高鸿山、慧海湾、雪浪小镇发展水平，办好2019世界物联网博览会；推动集成电路产业做大做强，加快延伸上下游产业链，着力构建核心产业集群和完整产业生态；促进先进制造业与现代服务业、先进制造业与新一代信息技术的深度融合，实施“千企技改”装备升级行动和100个智能制造重点项目，重点培育一批工业互联网平台，工业技改投资增长8%左右；　加快发展现代物流、工业设计、金融服务等现代服务业，推进梁溪区商贸流通创新发展示范区建设，支持无锡国家数字电影产业园创建中国电影创新试验区，继续办好无锡“文博会”、文化创意设计大赛等文化产业创新平台。落实“一城一策”要求，保持房地产市场持续平稳健康发展。

　　扩大军民融合影响力。争创国家军民融合创新示范区，加快军民融合公共服务平台“一网四中心”建设。聚焦航空发动机及燃气轮机、物联网、海洋工程装备等重点领域，鼓励民营企业积极参与国防建设、承接军转民技术转移，支持军工领域优势企业和科研院所在锡设立区域性总部，促进军民技术相互支撑、有效转化。

　　（三）着力提升科技创新能力

　　完善区域科技创新体系。围绕苏南国家自主创新示范区建设目标，加快推进国家物联网创新中心、国家高性能计算技术创新中心、国家集成电路特色工艺及封装测试创新中心等重大平台建设。强化与驻锡科研院所“一所一策”合作，推动与本土企业紧密对接，加快技术成果转化。布局新一轮创新创业孵化载体建设，提升现有科技载体发展水平，新增省级以上众创空间5家以上。大力吸引国际高端创新机构、跨国公司研发中心、国际科技合作组织来锡发展。积极创建国家知识产权强市，万人有效发明专利拥有量达到39件。

　　加快高新技术产业化。引导企业加大研发投入，新增各级各类研发机构80家以上，企业研发经费占销售收入比重达到1.74%。加大雏鹰企业、瞪羚企业、准独角兽企业培育力度，全市高新技术企业达到2600家，高新技术产业产值占规模以上工业产值比重提高到44%。加强与国内外著名高校院所的合作，新增院士工作站7家以上。实施产业前瞻与关键共性技术研发计划和重大成果转化计划，办好2019无锡市产学研合作科技成果洽谈会。

　　培育和集聚各类优秀人才。深入实施“太湖人才计划”升级版2.0和名校名院名所名企“四名工程”，继续办好无锡（太湖）高层次人才创新创业交流大会、“百企千才高校行”等活动，加快构建“一站式”人才服务平台，高水平打造一流人才生态环境，全年新增各类人才7万人，其中高层次人才0.7万人、高技能人才1.35万人。

　　（四）着力实施乡村振兴战略

　　加快发展现代农业。强化农业基础设施建设和粮食安全保障工作，提升农业生产装备水平和社会化服务能力，全市高标准农田比重达到80%以上，农业机械化水平达到90%。调整优化农业生产结构，提高优质绿色农产品比重，培育壮大“锡”字号农产品品牌。扎实推进“百企建百园”工程，提高国家级和省级现代农业产业示范园区建设水平，农业园区面积比重达到52%。大力培育农业新型经营主体，新增市级以上农业龙头企业6家，扶持农民专业合作社20家，市级以上示范家庭农场增加到170家。促进农村一二三产业深度融合，加快发展“农业+互联网+快递”“农业+旅游”等新业态。

　　深化农村改革创新。加快农村承包地、宅基地“三权分置”和农村土地承包经营权抵押贷款试点，依法依规开展土地流转。探索激活集体经营性建设用地的政策措施，进一步扶持壮大农村集体经济。深化农村集体产权制度改革，扎实推进集体经济股份制改革。完善财政支农投农机制，推动农村金融制度改革，带动更多社会资本参与乡村建设发展。落实供销合作综合改革任务。

　　高质量推进乡村建设。加快村庄规划全覆盖，有序推进农村住房更新改造，新建美丽乡村示范村25个、美丽乡村休闲旅游示范村8个。强化“四好农村路”建设，新建改建农村道路58公里、桥梁31座。开展农村人居环境整治提升行动，建设100个美丽宜居村庄，创建100个村庄环境长效管理示范村。扎实推进“大棚房”问题专项清理整治。力争全面完成经济薄弱村脱困转化任务。

　　（五）着力打好污染防治攻坚战

　　扎实推进蓝天、碧水、净土保卫战。深化太湖流域工业污染、农业面源污染、入湖河流等综合整治，推进太湖生态清淤试点，开展河湖违法圈圩、违法建设专项整治，推动太湖无锡水域水质持续改善，确保国省考断面优III比例达到省下达目标。加大大气污染防治力度，推进机动车污染整治，强化施工扬尘管理，确保PM2.5平均浓度持续下降、空气优良天数比率持续上升。强化土壤污染防治，推进企业用地土壤污染状况调查，落实污染地块环境管理联动机制，确保土壤安全利用。继续深入推进“绿刃”环保专项行动，加大行政执法与司法联动力度，提高环境监管和执法水平。

　　进一步夯实绿色发展基础。开展国家级生态保护红线划定和省生态保护红线校核工作，严格执行生态红线保护措施。深入推进节能降耗，全市单位GDP能耗降幅完成省下达目标任务。加快化工钢铁煤电行业转型升级，进一步加大“散乱污”企业整治力度，多措并举减少污染物排放。有序推进园区循环化改造，组织实施节能与循环经济项目80个，新增国家级绿色工厂2家。做好锡东、惠联垃圾焚烧电厂提标扩能工作，积极推进餐厨垃圾、蓝藻藻泥、市政污泥、酸洗污泥等固废处置设施建设，力争早日建成投运。

　　持续提高生态文明建设水平。抓好中央环保督察“回头看”等反馈问题整改工作。加快转变沿太湖地区发展模式，打造太湖生态保护圈；落实“共抓大保护，不搞大开发”要求，建设江阴长江生态安全示范区；统筹山水林田湖草系统治理，推进宜兴生态保护引领区建设。提高环境污染责任保险工作水平，推动形成以排污许可证为核心的环保监管体系。不断加强领导干部自然资源资产离任审计。

　　（六）着力提升城市能级和枢纽功能

　　建设全国性综合交通枢纽。围绕长三角区域枢纽机场定位，编制完成苏南硕放机场总体规划和集疏运体系规划，启动机场停机坪、新货站扩建，持续增加国际客货运航线，加快药品、生物制品、冰鲜水产品等进口指定口岸建设，不断提升口岸等级和能力，争取全年旅客吞吐量达到770万人次、货邮吞吐量达到14万吨。围绕完善铁路枢纽功能，加快南沿江铁路建设，做好盐泰锡常宜铁路前期工作。围绕发挥区域性物流枢纽作用，全力抓好苏锡常南部高速等项目建设，积极推进锡宜高速、沿江高速扩建和锡太高速等项目前期工作，力争《无锡（江阴）港总体规划》早日批复，进一步提升高新空港物流园、西站物流枢纽等货运枢纽发展水平。

　　加快锡澄锡宜一体化发展。推进国土空间规划编制工作。支持江阴打造长江下游滨江新兴中心城市、宜兴打造宁杭生态经济带新兴中心城市。开工建设锡澄城际轨道S1线、宜马快速通道、宜兴丁蜀通用机场等项目，续建凤翔路快速化改造等项目，推进锡宜城际轨道S2线、暨南大道西延等项目前期工作，确保锡澄锡宜基础设施一体化建设取得关键性进展。编制完成锡澄、锡宜协同区规划，优化提升惠山新城、太湖国家旅游度假区功能定位，鼓励和引导重大产业项目向江阴南部、宜兴东北部等重点协作区转移落地。

　　提升中心城市集聚辐射能力。全面接轨上海、服务上海，加强与苏州、常州的规划对接，推动基础设施互联互通。深化“一城两核”定位，加快环城河以内历史城区“双修”工作，持续推进棚户区改造，提升中山路黄金商圈影响力；启动太湖新城新一轮建设发展，组织实施太湖新城绿色可持续发展与国际合作、无锡国际会议中心、高浪路快速化改造等项目。强化锡东新城区域支点地位，精心谋划蠡湖新城开发建设。启动第三轮轨道交通建设规划编制，加快地铁3号线、4号线一期工程建设，地铁1号线南延线年底开通试运营。启动312国道快速化改造、菱湖大桥等重点道桥项目，打通一批“断头路”，疏通一批“卡脖子路”。

　　建设更高水平的智慧城市。组织实施新型智慧城市建设三年行动计划，加快城市大数据中心建设，健全大数据管理运营机制，深入推进城市数据资源的全面共享和广泛运用。构建智慧城市移动门户，汇集各类民生服务应用。加快5G商用网络建设和下一代互联网的规模化部署，促进城市信息基础设施跨越式发展。

　　（七）着力加强城市管理和社会治理

　　提升城市精细化管理水平。大力开展优美环境合格区建设，扎实推进七大专项提升行动，更高水平建设智慧城管项目，有效解决违法建设、“三乱”违法广告、农贸市场脏乱差、“小飞龙”等一批城市管理热点难点问题。全面推进生活垃圾分类工作，提高分类质量，垃圾分类集中处理率达到75%。健全文明城市创建长效机制，实施《无锡市文明行为促进条例》，提高市民素养和全社会文明程度。

　　推进城市安全发展。落实完善城市运行管理及相关方面的安全生产责任制，进一步健全公共安全体系，持续强化防灾减灾救灾能力，全面提高城市安全保障水平。加快新孟河、望虞河西岸控制和除险加固工程、锡澄运河北排扩大工程等项目建设，整体提升防洪排涝能力。积极配合国家特高压工程建设，打造现代城市高端智能电网。坚持发展新时代“枫桥经验”，推动警务综治一体化，加快立体化、信息化社会治安防控体系建设，启动实施“雪亮工程”二期和“慧眼工程”，全面深化扫黑除恶专项斗争，争创平安中国示范区。强化市场监督综合管理，守好食品药品和产品质量安全防线。

　　深化基层治理创新。优化城乡社区治理体制机制，健全网格化社会治理机制，加强社区工作者队伍建设，进一步减轻村（社区）负担。做好新一届村（居）民委员会换届选举工作，加强村务公开和民主管理，完善“一核心四平台”乡村治理体系。构建三级信访工作联席会议体系，增强矛盾化解和源头预防能力。完善公共法律服务体系，加强“社区矫正服务志愿者”队伍建设。加大向社会组织购买公共服务力度，提升社会组织发展水平。

　　（八）着力保障和改善民生

　　推动实现高质量就业。实施积极的就业政策，推进全民创业行动计划，促进大学生等重点群体就业创业，强化对就业困难人员的分类帮扶，努力化解就业结构性矛盾，办好首届“创响无锡”全民创业大赛等活动，确保新增城镇就业11万人。落实个人所得税改革和基本工资标准正常调整机制，进一步提升居民经营性与财产性收入，实现富民增收与经济发展同步、劳动报酬与生产效率提高同步。

　　加强社会保障体系建设。持续提高低保、临时救助、残疾人等保障标准，开工建设儿童福利院异地重建等项目，加快市残疾人综合服务基地项目建设。深入推进养老服务“双试”改革，新建智慧养老服务机构30家以上，养老护理床位占比达56%以上，接受上门服务的居家老人数占比达到10%。制定住房保障中长期发展规划，进一步提高公租房运营管理水平。做好退役军人工作，进一步提高双拥共建、军转安置、优抚工作质量。

　　推进教育改革发展。提高学前教育资源配置率和优质率，加快义务教育学校标准化建设，积极创建省高品质示范高中。实施职业教育现代化工程，优化职业院校布局和专业设置，完善无锡职教园管理体制。支持江南大学建设世界一流学科，持续深化与江阴、宜兴的办学合作。加快东南大学国家示范性微电子学院、南京信息工程大学滨江学院无锡校区二期工程、南京理工大学江阴校区建设。支持无锡太湖学院建设高水平应用型本科大学，支持具备条件的高职院校建设本科层次职业学校。积极筹建无锡师范高等专科学校。

　　补齐医疗卫生服务短板。推进全国健康城市试点工作，新建成省级健康镇村（社区）50个、健康单位55个以上。推进市属医疗卫生机构布局调整优化重大项目建设，建成启用江南大学附属医院南院区，完成市妇幼保健院妇保、儿保大楼建设。做优做强县级（区级）医院，提升城区紧密型医联体和县域医共体建设水平，推动基层医疗卫生机构提档升级，完善分级诊疗制度。持续深化综合医改，加快建立现代医院管理制度，推进江阴、宜兴城乡居民医保制度整合，深化疾病诊断相关分组付费试点工作，复制推广村级医疗互助机制。制定紧缺专学科发展规划，加大卫生人才引进、培养、管理力度，进一步提升江南大学无锡医学院建设水平。

　　提升城市文化旅游体育软实力。保护传承江南文脉，推进大运河文化带规划建设，与扬州联合办好世界运河大会，做好惠山古镇联合申遗筹备工作。高标准推进无锡美术馆建设，支持无锡博物院争创国家一级馆。积极创建省书香城市示范市，提升居民综合阅读率。深化国家全域旅游示范区创建，推进旅游公共服务体系建设，优化省级以上旅游度假区组织领导和管理体制，加快无锡融创文旅城等重大旅游项目建设。积极争创全国全民运动健身模范市，继续办好无锡国际马拉松等一批大型国际赛事。

　　各位代表，今年我们将继续围绕人民群众普遍关心的热点难点问题，进一步整合资源，加大投入力度，扎实办好10件民生实事，主要内容有：一是污染防治项目。巩固市区黑臭河道整治成果，确保建成区全面消除黑臭水体。启动锡澄片骨干河网畅流活水工程。惠联餐厨废弃物处理厂建成投运。二是帮扶济困项目。实施长期护理保险制度、市区中低收入居民疾病医疗自费支出救助制度和“关爱圆梦”希望工程助学项目。全市经济薄弱村脱困转化7个。三是重点道桥项目。完成运河东路大修工程，开工建设新锡澄路北延、华谊路、稻香路、新北桥拆危建新等工程。四是公共交通项目。新辟和优化调整公交线路10条以上，新增、更新新能源公交车和低碳环保公交车260辆。五是宜居住区项目。完成88万平方米宜居住区（旧住宅区）建设和50万平方米棚户区（危旧房、城中村）改造。市区新增机动车停车泊位5万个，完成二次供水设施改造项目50个，改造老旧自来水管网50公里，新增天然气用户5万户，新增城市绿地面积150万平方米。完成城区环卫公厕改建30座。六是食品安全项目。完成无锡市食品安全检验检测中心建设。“互联网+明厨亮灶”建设覆盖1万家餐饮服务单位。市区完成农贸市场改造6家，实现规模型农贸市场远程智慧监管全覆盖。七是智慧城市项目。光纤网络户均带宽达到每秒120兆比特，新建5G基站500个。全市4A级以上景区实现智能导览，300个热点公共区域实现智能监管。城市交通出行实现“一码通”，机场实现无纸化登机，30%以上公共停车位信息接入智慧停车平台，车联网城市级示范应用项目用户数达到10万。建设办税、缴费“网上办、一次办、就近办”服务体系。八是文化建设项目。建设200个新时代文明实践中心（所、站）。完成“文艺进万家”活动150场次。打造古运河健身步道和西蠡湖、贡湖湾健身路径。九是卫生事业项目。免费为1和4周岁儿童接种水痘疫苗15万人次，免费为65—85岁户籍老年人接种肺炎球菌疫苗。十是教育事业项目。实施中小学课后延时服务制度，开工义务教育学校基建项目28个、幼儿园基建项目13个。

　　三、持续加强政府自身建设

　　各位代表，做好今年工作，关键在于扎实推进政府自身建设。我们将深入贯彻落实新时代党的建设总要求，坚持以习近平新时代中国特色社会主义思想武装头脑、指导实践、推动工作，树牢“四个意识”，坚定“四个自信”，做到“两个坚决维护”，自觉在思想上政治上行动上同以习近平同志为核心的党中央保持高度一致，切实把党对一切工作的领导贯彻到政府工作各领域各方面，进一步提高施政能力和服务水平，为圆满完成全年目标任务提供坚强保证。

　　（一）聚焦责任政府建设，加快转变政府职能。按照市委统一部署，完成政府机构改革任务，着力构建系统完备、科学规范、运行高效的政府机构职能体系。深化市区两级政府财政事权和支出责任划分改革，强化中期财政规划对年度预算的约束，全面加强预算绩效管理。健全抓落实的指标体系、工作体系和评价体系，少发不管用不实用的文件，少开没有实际效果的会议。落实政府性债务化解责任，确保存量不出险、增量不违规、有效保障发展。

　　（二）聚焦法治政府建设，深入推进依法行政。加强社会民生、城市管理等重点领域的政府立法工作，强化政府规章和规范性文件的合法性审查、后评估和即时清理。健全重大行政决策体制机制，确保重大事项议深议透、形成各方共识，着力提高政府决策的科学化、民主化、法治化水平。认真执行市人大及其常委会的决议决定，落实向市人大常委会报告国有资产管理情况制度，主动接受市政协民主监督，加强司法、舆论、社会监督，确保权力在阳光下运行。

　　（三）聚焦服务政府建设，切实提高运转效能。加强政府部门目标管理，建立健全跨区域、跨部门的目标执行和评估体系，加大对重大政策措施贯彻落实情况的督查力度。加快全市政务服务“一体化”建设，推进“一窗式”改革，实现更加智慧的网上办理能力。加大政府热线整合力度，建立政府热线联动机制，做到企业和群众诉求“一号响应”。积极运用鼓励激励、容错纠错、能上能下“三项机制”，推动政府系统形成真抓实干、奋勇争先的良好局面。

　　（四）聚焦廉洁政府建设，坚决扎紧制度笼子。认真履行党风廉政建设“一岗双责”，扎实做好中央巡视反馈意见整改工作，落实中央八项规定及其实施细则精神和省、市委具体办法，狠抓办公用房清理和公务用车管理常态化工作，市本级一般性支出比去年压减10%。整合公共资源交易平台，加快实现“不见面”开标。加大重点领域审计和监管力度，做到应审尽审、凡审必严、严肃问责。坚决防止和反对腐败，着力解决人民群众身边的不正之风和腐败问题。

　　各位代表，行人莫道春来早，又是乘风远济时。让我们更加紧密地团结在以习近平同志为核心的党中央周围，在中共无锡市委的坚强领导下，紧紧依靠全市广大人民，不忘初心、牢记使命，艰苦创业、埋头实干，团结拼搏、开拓创新，为打好高水平全面建成小康社会决定性基础、夺取“强富美高”新无锡建设关键性胜利、当好全省高质量发展领跑者而努力奋斗！
